# The Burden of Alcohol Use: Excessive Alcohol Consumption and Related Consequences Among College Students

**DOI:** 10.35946/arcr.v35.2.11

**Published:** 2014

**Authors:** Aaron White, Ralph Hingson

**Affiliations:** **Aaron White, Ph.D.**, *is program director, College and Underage Drinking Prevention Research; and*; **Ralph Hingson, Sc.D.**, *is director, Division of Epidemiology and Prevention Research, both at the National Institute on Alcohol Abuse and Alcoholism, Bethesda, Maryland.*

**Keywords:** Alcohol consumption, alcohol use, abuse, and dependence, alcohol burden, alcohol effects and consequences, harmful drinking, underage drinking, binge drinking, college student, risk factors, genetic factors, environmental factors, social norms, parental attitude, Greek organization, athletes, community environment, academic performance, injury, sexual assault, overdose, memory blackout, brain function, cognitive deficits, death, accessibility, availability, affordability, survey, data collection, data analysis

## Abstract

Research shows that multiple factors influence college drinking, from an individual’s genetic susceptibility to the positive and negative effects of alcohol, alcohol use during high school, campus norms related to drinking, expectations regarding the benefits and detrimental effects of drinking, penalties for underage drinking, parental attitudes about drinking while at college, whether one is member of a Greek organization or involved in athletics, and conditions within the larger community that determine how accessible and affordable alcohol is. Consequences of college drinking include missed classes and lower grades, injuries, sexual assaults, overdoses, memory blackouts, changes in brain function, lingering cognitive deficits, and death. This article examines recent findings about the causes and consequences of excessive drinking among college students relative to their non-college peers and many of the strategies used to collect and analyze relevant data, as well as the inherent hurdles and limitations of such strategies.

Since 1976, when the National Institute on Alcohol Abuse and Alcoholism (NIAAA) issued its first report on alcohol misuse by college students, research advances have transformed our understanding of excessive drinking on college campuses and the negative outcomes that follow from it. For instance, we now know that a broad array of factors influence whether a particular college student will choose to drink, the types of consequences they suffer from drinking, and how they respond to those consequences. We have learned that predisposing factors include an individual’s genetic susceptibility to the positive and negative effects of alcohol, alcohol use during high school, campus norms related to drinking, expectations regarding the benefits and detrimental effects of drinking, penalties for underage drinking, parental attitudes about drinking while at college, whether one is member of a Greek organization or involved in athletics, and conditions within the larger community that determine how accessible and affordable alcohol is. Consequences include missed classes and lower grades, injuries, sexual assaults, overdoses, memory blackouts, changes in brain function, lingering cognitive deficits, and death.

This article reviews recent research findings about alcohol consumption by today’s college students and the outcomes that follow. It examines what we know about the causes and consequences of excessive drinking among college students relative to their non-college peers and many of the strategies used to collect and analyze relevant data, as well as the inherent hurdles and limitations of such strategies.

## Excessive Drinking At College

Currently, only two active national survey studies are able to characterize the drinking habits of college students in the United States. The National Survey on Drug Use and Health (NSDUH), an annual survey sponsored by the Substance Abuse and Mental Health Services Administration (SAMHSA), involves face-to-face interviews with approximately 67,500 persons ages 12 and older each year regarding use of alcohol and other drugs. Monitoring the Future (MTF) is an annual, paper-and-pencil national survey of alcohol and other drug use with a sample comprising nearly 50,000 students in 8th, l0th, and 12th grades drawn from roughly 420 public and private schools. Approximately 2,400 graduating seniors are resurveyed in subsequent years, allowing for the monitoring of trends in college drinking.

In addition, two prior surveys yielded data on college drinking that remain valuable and relevant. The National Epidemiologic Survey on Alcohol and Related Conditions (NESARC), sponsored by NIAAA, collected data on alcohol and other drug use from a sample of roughly 46,500 citizens 18 and older using face-to-face computer-assisted interviews. Two waves of data (2001 and 2004) were collected from the same sample, and data from an independent sample are scheduled to be collected in 2013. The Harvard College Alcohol Study (CAS), although no longer active, was a landmark paper-and-pencil survey that provided national data (years 1993, 1997, 1999, and 2001) from roughly 15,000 students on more than 100 college campuses each year (Wechsler and Nelson 2008). Data from both NESARC and Harvard CAS remain useful for examining associations between patterns of drinking at college and the frequency and prevalence of alcohol-related consequences for both drinkers and nondrinkers.

**Figure 1:**
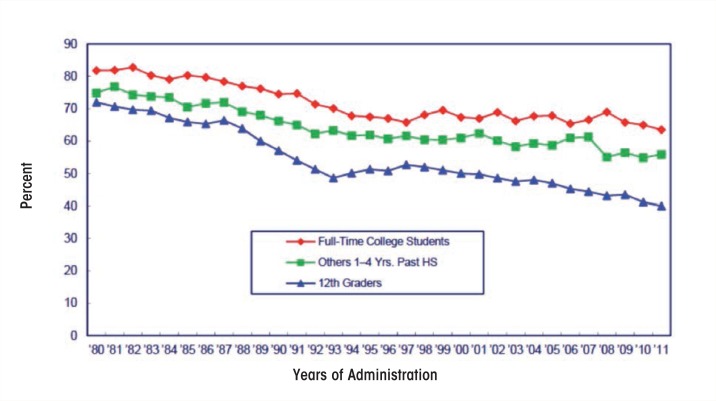
Alcohol: Trends in 30-day prevalence among college students vs. others 1 to 4 years beyond high school (twelfth graders included for comparision). SOURCE: The Monitoring the Future Study, the University of Michigan. NOTE: Others refers to high school graduates 1 to 4 years beyond high school not currently enrolled full time in college.

Data from NSDUH and MTF suggest that roughly 65 percent of college students drink alcohol in a given month (see figure 1 for data from MTF), and Harvard CAS all suggest that a large percentage of college students who drink do so to excess. Excessive, or “binge,” drinking is defined in NSDUH, MTF, and NESARC as consuming five or more drinks in an evening, although the instruments vary in the specified time frames given (i.e., once or more in the past month for NSDUH, past 2 weeks for MTF, and multiple time periods for NESARC) (Johnston et al. 2001*a*; SAMHSA 2011). The Harvard CAS was the first national study of college students to utilize a gender-specific definition of binge drinking (i.e., four or more drinks in an evening for females or five or more for males in the past 2 weeks) to equate the risk of alcohol-related harms (Wechsler et al. 1995). The Centers for Disease Control and Prevention (CDC) utilizes the same four or more/five or more gender-specific measures but specifies a 30-day time period (Chen et al. 2011). NIAAA uses the four or more/five or more gender-specific measure but specifies a time frame of 2 hours for consumption, as this would generate blood alcohol levels of roughly 0.08 percent, the legal limit for driving, for drinkers of average weight (NIAAA 2004).

**Figure 2:**
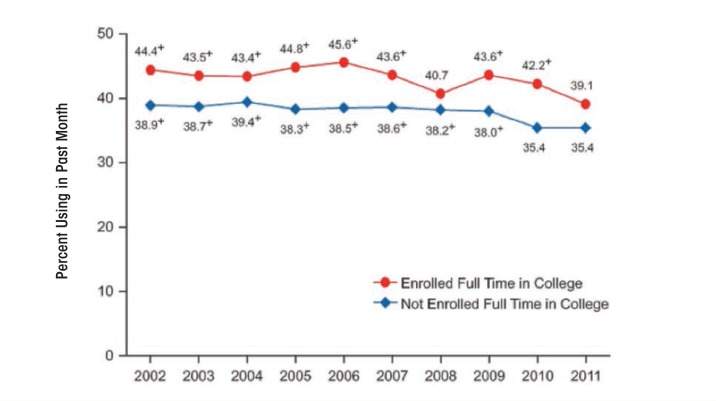
Binge alcohol use among adults aged 18 to 22, by college enrollment: 2002–2011. Survey years are shown on the horizontal axis, and the percentage using in the past month is shown on the vertical axis. For each college enrollment status (enrolled full time in college and not enrolled full time in college), there is a line showing use over the years 2002, 2003, 2004, 2005, 2006, 2007, 2008, 2009, 2010, and 2011. Tests of statistical significance at the .05 level were performed between 2011 and each of the previous years listed; significant results are indicated where appropriate. Among adults aged 18 to 22 enrolled full time in college, 44.4 percent were past-month binge alcohol users in 2002, 43.5 percent in 2003, 43.4 percent in 2004, 44.8 percent in 2005, 45.6 percent in 2006, 43.6 percent in 2007, 40.7 percent in 2008, 43.6 percent in 2009, 42.2 percent in 2010, and 39.1 percent in 2011. The differences between the 2011 estimate and the 2002, 2003, 2004, 2005, 2006, 2007, 2009, and 2010 estimates were statistically significant. Among adults aged 18 to 22 not enrolled full time in college, 38.9 percent were past-month binge alcohol users in 2002, 38.7 percent in 2003, 39.4 percent in 2004, 38.3 percent in 2005, 38.5 percent in 2006, 38.6 percent in 2007, 38.2 percent in 2008, 38.0 percent in 2009, 35.4 percent in 2010, and 35.4 percent in 2011. The differences between the 2011 estimate and the 2002 through 2009 estimates were statistically significant. SOURCE: Substance Abuse and Mental Health Services Administration. *Results From the 2011 National Survey on Drug Use and Health: Summary of National Findings,* NSDUH Series H–44, HHS Publication No. (SMA) 12–4713. Rockville, MD: Substance Abuse and Mental Health Services Administration, 2012.

According to NSDUH, the percentage of 18- to 22-year-old college students who reported drinking five or more drinks on an occasion in the previous 30 days remained relatively stable from 2002 (44 percent) to 2010 (44 percent) (SAMHSA 2011). Among 18- to 22-year-olds not enrolled in college, the percentage who engaged in binge drinking decreased significantly from 2002 (39 percent) to 2010 (36 percent) (see figure 2).

**Figure 3:**
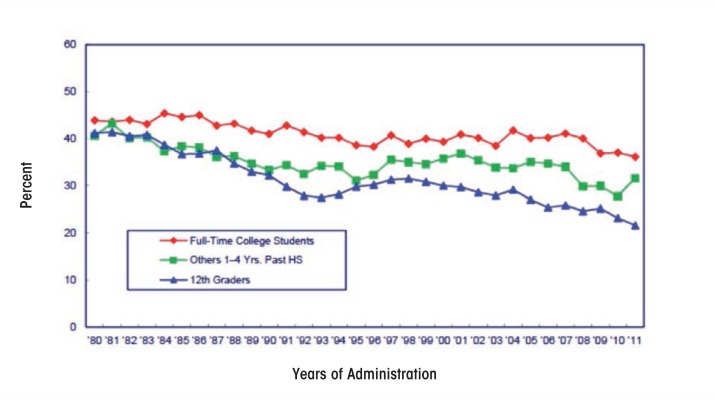
Alcohol: Trends in 2-week prevalence of consuming five or more drinks in a row among college students vs. others 1 to 4 years beyond high school (12th graders included for comparision). SOURCE: The Monitoring the Future study, the University of Michigan. NOTE: Others refers to high school graduates 1 to 4 years beyond high school not currently enrolled full time in college.

Looking at a longer time period, data from MTF suggest that there have been significant declines in the percentage of college students consuming five or more drinks in the previous 2 weeks, from 44 percent in 1980 to 36 percent in 2011 (Johnston et al. 2012) (see figure 3). This time frame includes the passage of the National Minimum Drinking Age Act of 1984, which effectively increased the drinking age from 18 to 21 in the United States.

**Figure 4:**
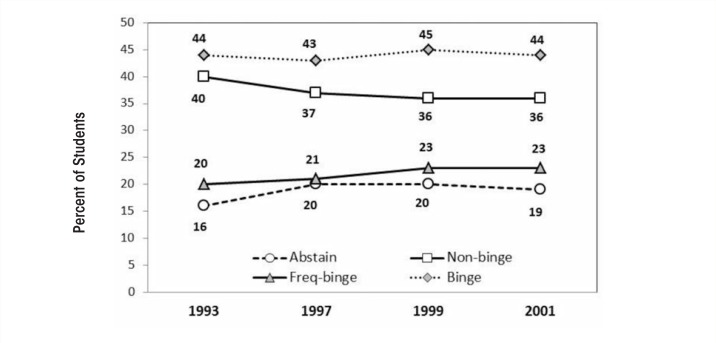
Drinking habits of college students from Harvard CAS. SOURCE: Johnston, L.D.; O’Malley, P.M.; Bachman, J.G.; and Schulenberg, J.E. *Monitoring the Future National Survey Results on Drug Use, 1975–2011: Volume I: Secondary School Students.* Ann Arbor, MI: Institute for Social Research, the University of Michigan.

Across the four waves of data collection in the Harvard CAS (1993, 1997, 1999, and 2001), rates of binge drinking remained relatively stable (44, 43, 45, and 44 percent, respectively) (Wechsler et al. 2002) (see figure 4). However, the number of non–binge drinkers decreased, whereas the number of frequent binge drinkers (three or more binge-drinking episodes in a 2-week period) increased. Wechsler and colleagues (2002) reported that binge drinkers consumed 91 percent of all the alcohol consumed by college students during the study period. Frequent binge drinkers, a group comprising only 1 in 5 college students, accounted for 68 percent of all alcohol consumed (Wechsler and Nelson 2008).

## Individual and Environmental Contributors to Excessive Drinking

**Figure 5:**
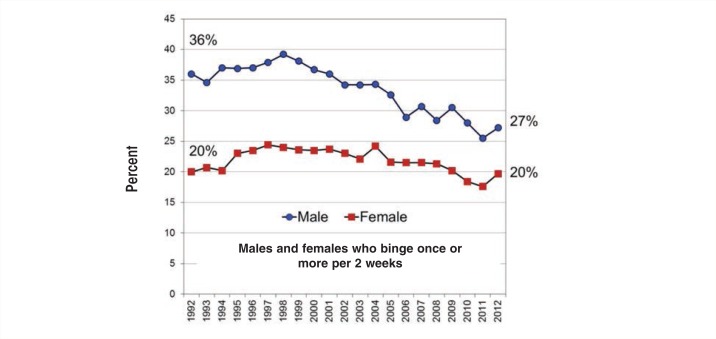
Percent of 12th-grade male and female students who reported drinking at least once in the prior 2 weeks. SOURCE: Wechsler, H.; Lee, J.E.; Kuo, M., et al. Trends in college binge drinking during a period of increased prevention efforts: Findings from 4 Harvard School of Public health College alcohol study surveys: 1993–2001. *Journal of American College Health* 50(5):203–217, 2002. PMID: 11990979

Survey data indicate that males outpace females with regard to binge drinking. According to MTF, in 2011, 43 percent of male and 32 percent of female college students crossed the binge threshold in a given 2-week period. Further, 40 percent of students—more males (44 percent) than females (37 percent)—reported getting drunk in a given month. Research suggests that gender differences in alcohol use by college students have narrowed considerably over the years. In their landmark 1953 report on college drinking, Yale researchers Straus and Bacon indicated that, based on survey data from more than 15,000 students on 27 college campuses, 80 percent of males and 49 percent of females reported having been drunk at some point. Nearly 60 years later, in 2011, data from MTF indicated that 68 percent of males and 68 percent females reported having been drunk. These new, higher levels of drinking among females seem to be ingrained in the youth drinking culture. Whereas binge-drinking rates declined significantly among high-school seniors over the last decade, the effect was driven by a decline among males only. Binge-drinking rates among females remained relatively stable (Johnston et al. 2012) (see figure 5).

Beyond gender, survey studies of college drinking reveal a range of characteristics of both individual students and campus environments that influence the likelihood of binge drinking. Data from the Harvard CAS and other studies reveal that males, Caucasians, members of Greek organizations, students on campuses with lower percentages of minority and older students, athletes, students coping with psychological distress, those on campuses near a high density of alcohol outlets, students with access to cheap drink specials, a willingness to endure the consequences of alcohol misuse, and drinking at off-campus parties and bars all contribute to excessive drinking (Mallett et al. 2013; Wechsler and Kuo 2003; Yusko et al. 2008). Further, students living off campus and/or in Greek housing, those who drink to try to fit it, students with inflated beliefs about the proportion of other students who binge drink, and those with positive expectations about the results of drinking are more likely to drink excessively (Scott-Sheldon et al. 2012; Wechsler and Nelson 2008). Importantly, excessive drinking prior to college relative to other college-bound students is predictive of both excessive drinking at college and experiencing alcohol-related consequences (Varvil-Weld et al. 2013; White et al. 2002).

## Strengths and Weaknesses of Binge-Drinking Measures

Several studies indicate that crossing commonly used binge-drinking thresholds increases a college student’s risk of experiencing negative alcohol-related consequences. For instance, data from the Harvard CAS indicate that students who binge one or two times during a 2-week period are roughly three times as likely as non–binge drinkers to get behind in school work, do something regretful while drinking, experience a memory blackout, have unplanned sex, fail to use birth control during sex, damage property, get in trouble with police, drive after drinking, or get injured (Wechsler et al. 2000). The more often a student binges, the greater the risk of negative outcomes. Further, the more binge drinking that occurs on a campus, the more likely non–binge drinkers and abstainers are to experience secondhand consequences of alcohol use, such as having studying or sleep disrupted, being a victim of sexual assault, and having property damaged (Wechsler and Nelson 2008).

Because of the increased risk of consequences to self and others that occurs when a person drinks at or beyond the binge threshold, a great deal of emphasis is placed on tracking the percentage of college students that cross binge thresholds. Although this has proven extremely valuable, as Wechsler and Nelson (2001, p. 289) state, “Alcohol use is a complex behavior. No single measure will capture all the relevant aspects of alcohol use.” One limitation of using a single threshold is that it removes data regarding just how heavily students actually drink (Alexander and Bowen 2004; Read et al. 2008) and assigns the same level of risk to all students who cross the thresholds regardless of how far beyond the threshold they go. This is an important consideration as recent studies suggest that plenty of college students who cross the binge threshold when they drink go far beyond it.

In a study of 10,424 first-semester college freshmen, more than one-half of all males and one-third of all females categorized as binge drinkers drank at levels two or more times the binge threshold (8 or more drinks for women and 10 or more drinks for men) at least once in the 2 weeks before the survey. Indeed, one in four binge-drinking males consumed 15 or more drinks at a time during that period (White et al. 2006). Naimi and colleagues (2010) reported that 18- 24-year-olds in the United States drink an average of 9.5 drinks per binge episode, nearly twice the standard binge threshold. Data from MTF also reveal that both college students and their non-college peers often drink at levels that exceed the binge threshold. On average, between 2005 and 2011, 7 percent of college females surveyed and 24 percent of college males consumed 10 or more drinks at least once in a 2-week period, compared with 7 percent of females and 18 percent of males not in college. Further, 2 percent of all college females surveyed and 10 percent of college males consumed 15 or more drinks in a 2-week period. Rates among non-college peers were similar, at 2 percent among females and 9 percent among males (Johnston et al. 2012). For a 140-pound female, consuming 15 drinks over a 6-hour period would produce an estimated blood alcohol level above 0.4 percent, a level known to have claimed, directly, several lives on college campuses in recent years. For a 160-pound male, drinking in this way would lead to a blood alcohol level above 0.3 percent, a potentially lethal level associated with memory blackouts and injury deaths.

Data from the Harvard CAS suggested that students who binge drink frequently (three or more times in a 2-week period) are at particularly high risk of negative alcohol-related outcomes. Compared with students who binge drink one or two times in a 2-week period, those who binge three or more times are twice as likely to experience alcohol-induced memory losses (27 percent vs. 54 percent, respectively), not use protection during sex (10 percent vs. 20 percent, respectively), engage in unplanned sex (22 percent vs. 42 percent, respectively), and get hurt or injured (11 percent vs. 27 percent, respectively), and are equally likely to need medical treatment for an overdose (1 percent vs. 1 percent). Whereas binge frequency is associated with an increased risk of negative outcomes, additional research indicates that there is a relationship between how often a student binges and the peak number of drinks he or she consumes. White and colleagues (2006) reported that 19 percent of frequent binge drinkers consume three or more times the binge threshold (12 or more drinks for females and 15 or more for males) at least once in a 2-week period compared with only 5 percent of infrequent binge drinkers. As a result of the association between frequency of binge drinking and peak levels of consumption, it is difficult to determine if the increase in risk that comes with frequent bingeing is a result of the number of binge episodes, per se, or the number of drinks consumed in an episode.

Importantly, although evidence suggests that many students drink at levels far beyond the binge threshold, additional research suggests that the majority of alcohol-related harms on college campuses result from drinking at levels near the standard four/five-drink measure. This is related to the well-known prevention paradox in which the majority of health problems, such as alcohol-related consequences, tend to occur among those considered to be at lower risk (Rose 1985). For a particular individual, the odds of experiencing alcohol-related harms increase as the level of consumption increases (Wechsler and Nelson 2001). However, at the population level, far fewer people drink in this manner. As a result, more total consequences occur among those who drink at relatively lower risk levels. For instance, based on data from roughly 9,000 college-student drinkers across 14 college campuses in California, Gruenewald and colleagues (2010) estimated that more than one-half of all alcohol-related consequences resulted from drinking occasions in which four or fewer drinks were consumed. Similarly, using national data from nearly 50,000 students surveyed across the four waves of the Harvard CAS, Weitzman and Nelson (2004) observed that roughly one-quarter to one-third of alcohol-related consequences, including getting injured, vandalizing property, having unprotected sex, and falling behind in school, occurred among students who usually consume three or four drinks per occasion. Such findings raise the possibility that a reduction in high peak levels of consumption might not necessarily result in large overall reductions in alcohol-related consequences on a campus. However, a reduction in high peak levels of drinking would certainly help save the lives of students who drink at these high levels.

In summary, while binge-drinking thresholds are useful for sorting students into categories based on levels of risk, a single threshold cannot adequately characterize the drinking habits of college students or the risks associated with alcohol use on college campuses (Read et al. 2008). It is not uncommon for college students to far exceed standard binge thresholds. Presently, only MTF tracks and reports the incidence of drinking beyond the binge threshold on college campuses. Such data are important as they allow for better tracking of changes in the drinking habits of students. For instance, it is possible that the number of students who drink at extreme levels could increase, whereas the overall percentage of students who binge drink declines or remains stable. Such a phenomenon might help explain why some consequences of excessive alcohol use, like overdoses requiring hospitalization, seem to be on the rise despite relatively stable levels of binge drinking on college campuses across several decades. Finally, although sorting students into binge drinking categories fails to capture high peak levels of consumption among students, a large proportion of harms actually occurs at or near the standard four or more/five or more threshold.

## Do Students Know How to Define Standard Servings?

Despite concerns about the accuracy of self-report data for assessing levels of alcohol use among college students and the general population, such surveys remain the most common tool for assessing alcohol use. One major concern is whether students and other young adults are aware of what constitutes a single serving of alcohol. Research shows that college students and the general public tend to define and pour single servings of alcohol that are significantly larger than standard drinks, suggesting they might underestimate their true levels of consumption on surveys (Devos-Comby and Lange 2008; Kerr and Stockwell 2012). For instance, White and colleagues (2003, 2005) asked students to pour single servings of different types of alcohol beverages into cups of various sizes. Overall, students poured drinks that were too large. When asked to simply define standard drinks in terms of fluid ounces, students tended to overstate the number of ounces in a standard drink. The average number of ounces of liquor in student-defined mixed drinks was 4.5 ounces rather than the 1.5 ounces in actual standard drinks (White et al. 2005). When students were provided with feedback regarding discrepancies between their definitions of single servings and the actual sizes of standard drinks, they tended to revise their self-reported levels of consumption upward, leading to a significant increase in the number of students categorized as binge drinkers (White et al. 2005). Such findings suggest that students underreport their levels of consumption on surveys, raising the possibility that more students drink excessively than survey data indicate.

Although a lack of knowledge regarding standard serving sizes could lead students to underestimate, and thus under-report, how much they drink, field research suggests that the discrepancy between self-reported and actual levels of consumption might be smaller than expected from lab studies. For instance, Northcote and Livingston (2011) conducted a study in which they monitored the number of drinks consumed by research participants in bars and then asked them to report their consumption a few days later. Reports by study participants were consistent with the observations made by researchers for participants who had consumed less than eight total drinks. Only those who consumed eight drinks or more tended to underestimate their consumption. When comparing estimated blood alcohol concentrations (BAC) based on self-report to actual BAC readings in college students returning to campus from bars, actual BAC levels tended to be lower, rather than higher, than levels calculated using self-reported consumption (Kraus et al. 2005). Similarly, when actual BAC levels are compared with estimated BAC levels in bar patrons, estimates are spread evenly between accurate, underestimates, and overestimates (Clapp et al. 2009).

In short, although self-reported drinking data might not be perfect, and college students lack awareness of how standard drink sizes are defined, research does not suggest that the discrepancies between self-reported and actual drinking levels are large enough to question the general findings of college drinking surveys.

## Paper-and-Pencil, Face-To-Face, and Electronic Surveys: Does It Make a Difference?

National surveys of college drinking often utilize paper-and-pencil questionnaires (e.g., MTF and Harvard CAS) or face-to-face computer-assisted personal interviews (e.g., NSDUH and NESARC). It now is possible to collect survey data electronically via the Internet and also using handheld devices, such as smartphones and personal digital assistants. This raises questions about the comparability between traditional survey methods and electronic data collection.

Several studies comparing traditional (e.g., paper and pencil) and electronic means of data collection suggest that the approaches yield generally similar results from survey participants (Boyer et al. 2002; Jones and Pitt 1999; LaBrie et al. 2006; Lygidakis et al. 2010). For instance, in a comparison of Web-based and paper-and-pencil survey approaches, Knapp and Kirk (2003) found no differences in outcomes, suggesting that Web-based surveys do not diminish the accuracy or honesty of responses. Similarly, LaBrie and colleagues (2006) observed similar outcomes of self-reported alcohol consumption in a paper-and-pencil survey and an electronic survey. However, other studies suggest that students actually feel more comfortable answering personal questions truthfully when completing questionnaires electronically (Turner et al. 1998), which can lead to higher levels of self-reported substance use and other risky behaviors. Both Lygidakis and colleagues (2010) and Wang and colleagues (2005) indicate that adolescents completing electronic surveys reported higher levels of alcohol and other drug use compared with those completing paper-and-pencil versions.

Response rate is an important consideration, with higher response rates increasing the representativeness of the sample and limiting the likelihood that response biases will influence the outcomes. Two national paper-and-pencil surveys mentioned above, MTF and Harvard CAS, report response rates for college students of approximately 59 percent. For MTF, this response rate represents a retention rate, as the participants were followed up after high school. Response rates for the in-person computer-assisted personal interviews, NSDUH and NESARC, which assess college student drinking but are not limited to college students, are roughly 77 percent and 81 percent, respectively. Currently, there is no basis for assessing response rates for national Web-based assessments of college drinking. However, smaller studies suggest that response rates might be comparable, if not higher, than other approaches. McCabe and colleagues (2002) reported that, among 7,000 undergraduate students, one-half of whom were surveyed about alcohol and other drug use via the Internet and half surveyed via paper-and-pencil surveys delivered through the mail, the response rates were 63 percent for the Web survey and 40 percent for the paper-and-pencil survey. Further, response rates for Web-based surveys can be improved by sending reminders via e-mail (van Gelder et al. 2010).

In summary, in recent years an increasing number of researchers have utilized electronic survey methods to collect college-drinking data. At present, evidence suggests that these methods can yield results quite similar to those obtained from traditional survey methods and that response rates might actually be higher.

## Alcohol-Related Consequences Among College Students

Drinking to intoxication leads to widespread impairments in cognitive abilities, including decisionmaking and impulse control, and impairments in motor skills, such as balance and hand-eye coordination, thereby increasing the risk of injuries and various other harms. Indeed, research suggests that students who report “getting drunk” even just once in a typical week have a higher likelihood of being injured, experiencing falls that require medical treatment, causing injury in traffic crashes, being taken advantage of sexually, and injuring others in various ways (O’Brien et al. 2006). Students who drink with the objective of getting drunk are far more likely to experience a range of consequences, from hangovers to blackouts, than other students who drink (Boekeloo et al. 2011).

National estimates suggest that thousands of college students are injured, killed, or suffer other significant consequences each year as a result of drinking. However, researchers have questioned the manner in which such national estimates are calculated. In many cases, the lack of college identifiers in datasets means that the actual amount of annual alcohol-attributable harm that occurs among college students is unknown. Although the Harvard CAS collected data regarding the consequences of drinking, its final year of administration was 2001. Currently, assessing the damage done, on a national level, by college drinking requires estimating rates of consequences using a variety of data sources. Such assessments are complicated by the fact that outcomes considered to be negative consequences by researchers (e.g., blackouts and hangovers) are not always perceived as negative by students (Mallett et al. 2013). Further, college students often drink off campus, such as during spring breaks and summer vacations, meaning that many alcohol-related consequences experienced by college students are not necessarily associated with college itself. As such, our understanding of alcohol-related consequences among college students remains somewhat cloudy.

In one set of estimates, Hingson and colleagues (2002, 2005, 2009) utilized census data and national datasets regarding traffic crashes and other injury deaths to estimate the prevalence of various alcohol-related harms among all young people aged 18–24. Next, they attributed an amount of harm to college students equal to the proportion of all 18- to 24-year-olds who were enrolled full time in 4-year colleges (33 percent in 2005, the most recent year analyzed) (Hingson et al. 2009). Because college students drink more heavily than their non-college peers, it is possible this approach underestimated the magnitude of alcohol-related consequences on college campuses. Hingson and colleagues (2002, 2005, 2009) also used the percentage of college students who reported various alcohol-related behaviors (e.g., being assaulted by another drinking college student) in national surveys to derive national estimates of the total numbers of college students who experienced these consequences.

Based on the above strategies along with other sources of data, researchers have estimated the following rates and prevalence of alcohol-related harms involving college students:

- **Death:** It is possible that more than 1,800 college students between the ages of 18 and 24 die each year from alcohol-related unintentional injuries, including motor-vehicle crashes (Hingson et al. 2009).
- **Injury:** An estimated 599,000 students between the ages of 18 and 24 are unintentionally injured each year under the influence of alcohol (Hingson et al. 2009).
- **Physical Assault:** Approximately 646,000 students between the ages of 18 and 24 are assaulted each year by another student who has been drinking (Hingson et al. 2009).
- **Sexual Assault:** Perhaps greater than 97,000 students between the ages of 18 and 24 are victims of alcohol-related sexual assault or date rape each year (Hingson et al. 2009).
- **Unsafe Sex:** An estimated 400,000 students between the ages of 18 and 24 had unprotected sex and nearly 110,000 students between the ages of 18 and 24 report having been too intoxicated to know if they consented to having sex (Hingson et al. 2002).
- **Health Problems:** More than 150,000 students develop an alcohol-related health problem each year (Hingson et al. 2002).
- **Suicide Attempts:** Between 1.2 and 1.5 percent of college students indicate that they tried to commit suicide within the past year as a result of drinking or drug use (Presley et al. 1998).
- **Drunk Driving:** Roughly 2.7 million college students between the ages of 18 and 24 drive under the influence of alcohol each year (Hingson et al. 2009).
- **Memory Loss:** National estimates suggest that 10 percent of non–binge drinkers, 27 percent of occasional binge drinkers, and 54 percent of frequent binge drinkers reported at least one incident in the past year of blacking out, defined as having forgotten where they were or what they did while drinking (Wechsler et al. 2000; White 2003).
- **Property Damage:** More than 25 percent of administrators from schools with relatively low drinking levels and more than 50 percent from schools with high drinking levels say their campuses have a “moderate” or “major” problem with alcohol-related property damage (Wechsler et al. 1995).
- **Police Involvement:** Approximately 5 percent of 4-year college students are involved with the police or campus security as a result of their drinking (Wechsler et al. 2002) and an estimated 110,000 students between the ages of 18 and 24 are arrested for an alcohol-related violation such as public drunkenness or driving under the influence (Hingson et al. 2002). A more recent national study reported that 8.5 percent of students were arrested or had other trouble with the police because of drinking (Presley and Pimentel 2006).
- **Alcohol Abuse and Dependence:** Roughly 20 percent of college students meet the criteria for an alcohol use disorder in a given year (8 percent alcohol abuse, 13 percent alcohol dependence). Rates among age mates not in college are comparable (17 percent any alcohol use disorder, 7 percent alcohol abuse, 10 percent alcohol dependence) (Blanco et al. 2008).

With regard to assessing the number of college students who die from alcohol each year, in addition to the lack of college identifiers in datasets, another barrier is the fact that levels of alcohol often are not measured in nontraffic fatalities. As such, attributable fractions, based on analyses of existing reports in which alcohol levels were measured postmortem, are used to estimate the number of deaths by various means that likely involved alcohol. The CDC often uses attributable fractions calculated by Smith and colleagues (1999) based upon a review of 331 medical-examiner studies. An updated approach is needed. The combination of including college identifiers in medical records and measuring alcohol levels in all deaths would allow for accurate assessments of the role of alcohol in the deaths of college students and their non-college peers.

## Academic Performance

About 25 percent of college students report academic consequences of their drinking, including missing class, falling behind in class, doing poorly on exams or papers, and receiving lower grades overall (Engs et al. 1996; Presley et al. 1996*a*, *b*; Wechsler et al. 2002). Although some published research studies have not found a statistically significant association between binge drinking and academic performance (Gill 2002; Howland et al. 2010; Paschall and Freisthler 2003; Williams 2003; Wood et al. 1997), studies linking binge drinking to poorer academic performance outnumber the former studies two to one. Presley and Pimentel (2006) reported that in a national survey of college students, those who engaged in binge drinking and drank at least three times per week were 5.9 times more likely than those who drank but never binged to perform poorly on a test or project as a result of drinking (40.2 vs. 6.8 percent), 5.4 times more likely to have missed a class (64.4 vs. 11.9 percent), and 4.2 times more likely to have had memory loss (64.2 vs. 15.3 percent) (Thombs et al. 2009). Singleton and colleagues (2007, 2009), in separate prospective studies, found negative associations between heavy alcohol use and grade point average. Jennison (2004), based on a national prospective study, reported binge drinkers in college were more likely to drop out of college, work in less prestigious jobs, and experience alcohol dependence 10 years later. Wechsler and colleagues (2000) and Powell and colleagues (2004), based on the Harvard CAS, found frequent binge drinkers were six times more likely than non–binge drinkers to miss class and five times more likely to fall behind in school. White and colleagues (2002) observed that the number of blackouts, a consequence of heavy drinking, was negatively associated with grade point average (GPA). It is important to note that although data regarding GPA often are collected via self-report, the negative association between alcohol consumption and GPA holds even when official records are obtained (Singleton 2007). Collectively, the existing research suggests that heavy drinking is associated with poorer academic success in college.

## Alcohol Blackouts

Excessive drinking can lead to a form of memory impairment known as a blackout. Blackouts are periods of amnesia during which a person actively engages in behaviors (e.g., walking, talking) but the brain is unable to create memories for the events. Blackouts are different from passing out, which means either falling asleep or becoming unconscious from excessive drinking. During blackouts, people are capable of participating in events ranging from the mundane, such as eating food, to the emotionally charged, such as fights and even sexual intercourse, with little or no recall (Goodwin 1995). Like milder alcohol–induced short-term memory impairments caused by one or two drinks, blackouts primarily are anterograde, meaning they involve problems with the formation and storage of new memories rather than problems recalling memories formed prior to intoxication. Further, short-term memory often is left partially intact. As such, during a blackout, an intoxicated person is able to discuss events that happened prior to the onset of the blackout and to hold new information in short-term storage long enough to have detailed conversations. They will not, however, be able to transfer new information into long-term storage, leaving holes in their memory. Because of the nature of blackouts, it can be difficult or impossible to know when a drinker in the midst of one (Goodwin 1995).

There are two general types of blackouts based on the severity of the memory impairments. Fragmentary blackouts, sometimes referred to as gray outs or brown outs, are a form of amnesia in which memory for events is spotty but not completely absent. This form is the most common. En bloc blackouts, on the other hand, represent complete amnesia for events (Goodwin 1995).

Blackouts surprisingly are common among college students who drink alcohol. White and colleagues (2002) reported that one-half (51 percent) of roughly 800 college students who had ever consumed alcohol at any point in their lives reported experiencing at least one alcohol-induced blackout, defined as awakening in the morning not able to recall things one did or places one went while under the influence. The average number of total blackouts in those who experienced them was six. Of those who had consumed alcohol during the 2 weeks before the survey was administered, 9 percent reported blacking out. Based on data from 4,539 inbound college students during the summer between high-school graduation and the start of the freshmen year, 12 percent of males and females who drank in the previous 2 weeks experienced a blackout during that time (White and Swartzwelder 2009). Data from the Harvard CAS indicate that blackouts were experienced in a 30-day period by 25 percent of students in 1993 and 27 percent of students in 1997, 1999, and 2001 (Wechsler et al. 2002). A small study by White and colleagues (2004), in which 50 students with histories of blackouts were interviewed, suggests that fragmentary blackouts are far more common than en bloc blackouts. Roughly 80 percent of students described their last blackout as fragmentary.

Blackouts tend to occur following consumption of relatively large doses of alcohol and are more likely if one drinks quickly and on an empty stomach, both of which cause a rapid rise and high peak in BAC (Goodwin 1995; Perry et al. 2006). For this reason, pregaming, or prepartying, which typically involves fast-paced drinking prior to going out to an event, increases the risk of blacking out. Labrie and colleagues (2011) reported that 25 percent of 2,546 students who engaged in prepartying experienced at least one blackout in the previous month. Playing drinking games and drinking shots were risk factors. Further, skipping meals to restrict calories on drinking days is associated with an increased risk of blackouts and other consequences (Giles et al. 2009).

Because blackouts typically follow high peak levels of drinking, it is not surprising that they are predictive of other alcohol-related consequences. Mundt and colleagues (2012) examined past-year blackouts in a sample of more than 900 students in a randomized trial of a screening and brief intervention for problem alcohol use and found that blackouts predicted alcohol-related injuries over a subsequent 2-year period. Compared with students who had no history of blackouts, those who reported one to two blackouts at baseline were 1.5 times more likely to experience an alcohol-related injury, whereas those with six or more blackouts were 2.5 times more likely. In a follow-up report based on the same sample, Mundt and Zakletskaia (2012) estimated that among study participants, one in eight emergency-department (ED) visits for alcohol-related injuries involved a blackout. On a campus of 40,000 students, this would translate into roughly $500,000 in annual costs related to blackout-associated ED visits.

In the study of 50 students with blackout histories by White and colleagues (2004), estimated peak BACs during the night of the last blackout generally were similar for males (0.30 percent) and females (0.35 percent), although it is unlikely that self-reported alcohol consumption during nights in which blackouts occur is highly accurate. A study of amnesia in people arrested for either public intoxication, driving under the influence, or underage drinking found that the probability of a fragmentary or en bloc blackout was 50/50 at a BAC of 0.22 percent and the probability of an en bloc blackout, specifically, was 50/50 at a BAC of 0.31 percent, based on breath alcohol readings (Perry et al. 2006). In their study of blackouts in college students, Hartzler and Fromme (2003*a*) noted a steep increase in the likelihood of blackouts above a BAC of 0.25 percent, calculated from self-reported consumption. Thus, from existing research, it seems that the odds of blacking out increase as BAC levels climb and that blackouts become quite common at BAC levels approaching or exceeding 0.30 percent. As such, the high prevalence of blackouts in college students points to the magnitude of excessive consumption that occurs in the college environment. It should be noted, however, that BAC levels calculated based on self-reported consumption are unlikely to be accurate given the presence of partial or complete amnesia during the drinking occasion.

It seems that some people are more sensitive to the effects of alcohol on memory than others and are therefore at increased risk of experiencing blackouts. Wetherill and Fromme (2011) examined the effects of alcohol on contextual memory in college students with and without a history of blackouts. Performance on a task was similar while the groups were sober, but students with a history of blackouts performed more poorly when intoxicated than those without a history of blackouts. Similarly, Hartzler and Fromme (2003*b*) reported that when mildly intoxicated, study participants with a history of blackouts performed more poorly on a narrative recall task than those without a history of blackouts. When performing a memory task while sober, brain activity measured with functional magnetic resonance imaging is similar in people with a history of blackouts and those without such a history (Wetherill et al. 2012). However, when intoxicated, those with a history of blackouts exhibit lower levels of activity in several regions of the frontal lobes compared with subjects without a history of blackouts.

Thus, studies suggest that there are differences in the effects of alcohol on memory and brain function between those who experience blackouts and those who do not. Research by Nelson and colleagues (2004), using data from monozygotic twins, suggests that there could be a significant genetic component to these differences. Controlling for frequency of intoxication, the researchers found that if one twin experienced blackouts, the other was more likely than chance to experience them as well. Further, Asian-American students with the aldehyde dehydrogenase ALDH2*2 allele^1^ are less likely to experience blackouts than those without it, even after adjusting for maximum number of drinks consumed in a day (Lucsak et al. 2006).

Several challenges hinder the assessment of blackouts and the events that transpire during them. Blackouts represent periods of amnesia. As such, it is difficult to imagine that self-reported drinking levels are highly accurate for nights when blackouts occur. Further, in order for a person to know what transpired during a blackout, and sometimes to be aware that a blackout occurred at all, they need to be told by other individuals. Often, the information provided by these other individuals is unreliable as they were intoxicated themselves (Nash and Takarangi 2011). Thus, it is quite likely that self-reported rates and frequencies of blackouts, drinking levels during nights in which blackouts occur, and the rates of various types of consequences that occur during them, are underestimated.

## Alcohol Overdoses

When consumed in large quantities during a single occasion, such as a binge episode, alcohol can cause death directly by suppressing brain stem nuclei that control vital reflexes, like breathing and gagging to clear the airway (Miller and Gold 1991). Even a single session of binge drinking causes inflammation and transient damage to the heart (Zagrosek et al. 2010). The acute toxic effects of alcohol in the body can manifest in symptoms of alcohol poisoning, which include vomiting, slow and irregular breathing, hypothermia, mental confusion, stupor, and death (NIAAA 2007*b*; Oster-Aaland et al. 2009). Using data from the Global Burden of Disease Study, the World Health Organization (WHO) estimated that, in 2002, alcohol poisoning caused 65,700 deaths worldwide, with 2,700 poisoning deaths occurring in the United States (WHO 2009). New stories about alcohol overdoses among college students and their non-college peers have become increasingly common, a fact that is perhaps not surprising given the tendency toward excessive drinking in this age-group.

To investigate the prevalence of hospitalizations for alcohol overdoses—which stem from excessive intoxication or poisoning—among college-aged young people in the United States, White and colleagues (2011) examined rates of inpatient hospitalizations for 18- to 24-year-olds between 1999 and 2008 using data from the Nationwide Inpatient Sample, which contains hospital discharge records from roughly 20 percent of all hospitals in the country. Hospitalizations for alcohol overdoses without any other drugs involved increased 25 percent among 18- to 24-year-olds from 1999 to 2008, highlighting the risks involved in heavy drinking. In total, nearly 30,000 young people in this age-group, more males (19,847) than females (9,525) were hospitalized for alcohol overdoses with no other drugs involved in 2008. Hospitalizations for overdoses involving other drugs but not alcohol increased 55 percent over the same time period, while those involving alcohol and drugs in combination rose 76 percent. In total, there were 59,000 hospitalizations in 2008 among 18- to 24-year-olds for alcohol overdoses only or in combination with other drugs. Given that 33 percent of people in this age-group were full-time college students at 4-year colleges in 2008, a conservative estimate would suggest approximately 20,000 hospitalizations for alcohol overdoses alone or in combination with other drugs involved college students, although the exact number is not known.

Data from the Drug Abuse Warning Network (DAWN) indicate that ED visits for alcohol-related events increased in a similar fashion as those observed for inpatient hospitalizations. Among those ages 18 to 20, ED visits for alcohol-related events with no other drugs increased 19 percent, from 67,382 cases in 2005 to 82,786 cases in 2009. Visits related to combined use of alcohol and other drugs increased 27 percent, from 27,784 cases in 2005 to 38,067 cases in 2009. In 2009, 12 percent of ED visits related to alcohol involved use of alcohol in combination with other drugs (SAMHSA 2011).

Alcohol interacts with a wide variety of illicit and prescription drugs, including opioids and related narcotic analgesics, sedatives, and tranquilizers (NIAAA 2007*a*; Tanaka 2002). Importantly, BAC required for fatal overdoses are lower when alcohol is combined with prescription drugs. An analysis of 1,006 fatal poisonings attributed to alcohol alone or in combination with other drugs revealed that the median postmortem BACs in those who overdosed on alcohol alone was 0.33 percent, compared with 0.13 percent to 0.17 percent among those who overdosed on a combination of alcohol and prescription drugs (Koski et al. 2003, 2005). The combined use of alcohol and other drugs peaks in the 18- to 24-year-old age range (McCabe et al. 2006), suggesting that college-aged young adults are at particularly high risk of suffering consequences from alcohol-and-other-drug combinations.

The above findings reflect the fact that heavy consumption of alcohol quickly can become a medical emergency. One does not need to get behind the wheel of a car after drinking or jump off a balcony into a swimming pool on a dare to risk serious harm. Simply drinking too much alcohol is enough to require hospitalization and potentially cause death. Further, combining alcohol with other drugs can increase the risk of requiring medical intervention substantially. Thus, efforts to minimize the consequences of alcohol-related harms on college campuses should not lose sight of the fact that alcohol often is combined with other drugs and, when this is the case, the risks can be greater than when alcohol or drugs are used alone.

Measuring the true scope of medical treatment for alcohol overdoses among college students is difficult for several reasons. First, in datasets such as the Nationwide Emergency Department Sample (NEDS) and the Nationwide Inpatient Sample (NIS), no college identifiers are included to indicate whether a young person treated for an alcohol overdose is enrolled in college. Many schools do not track or report the number of students treated for an alcohol overdose, and many students drink excessively when away from campus. Further, schools that implement Good Samaritan or Amnesty policies, which allow students to get help for overly intoxicated peers without fear of sanctions, could create the false impression that overdoses are on the rise. For instance, after Cornell University implemented an amnesty policy, they witnessed an increase in calls to residence assistants and 911 for help dealing with an intoxicated friend (Lewis and Marchell 2006). Given the dangerous nature of alcohol overdoses, with or without other drugs involved, it is important to improve the tracking of these events at colleges and in the larger community.

## Sexual Assault

Sexual assault is a pervasive problem on college campuses, and alcohol plays a central role in it. A study of roughly 5,500 college females on two campuses revealed that nearly 20 percent experienced some form of sexual assault while at college (Krebs et al. 2009). Data from the Harvard CAS suggested that 5 percent of women surveyed were raped while at college (Mohler-Kuo et al. 2004). In a national sample of students who completed the Core Alcohol and Drug Survey in 2005, 82 percent of students who experienced unwanted sexual intercourse were intoxicated at the time. Similarly, nearly three-quarters (72 percent) of respondents to the Harvard CAS study who reported being raped were intoxicated at the time. In many cases, rape victims are incapacitated by alcohol. In one study, 3.4 percent of rape victims reported being so intoxicated they were unable to consent (Mohler-Kuo et al. 2004). In a study of 1,238 college students on three campuses over a 3-year period, 6 percent of students reported being raped while incapacitated by alcohol (Kaysen et al. 2006).

Research suggests that the involvement of alcohol increases the risk of being victimized in several ways, such as by impairing perceptions that one is in danger and by reducing the ability to respond effectively to sexual aggression (Abbey 2002; McCauley et al. 2010; Testa and Livingston 2009). Further, alcohol might increase the chances that a male will commit a sexual assault by leading them to misinterpret a female’s friendly gestures or flirtation as interest in sex and by increasing sexual aggression (Abbey 2002). When asked to read a story about a potential date rape involving intoxicated college students, both male and female subjects who are intoxicated were more likely to view the female as sexually aroused and the male as acting appropriately (Abbey et al. 2003).

It is widely held that sexual assaults, with and without alcohol involvement, are underreported on college campuses. Title IX of the Education Amendments Act of 1972, a Federal civil rights law, requires universities to address sexual harassment and sexual violence. However, universities vary with regard to how they handle such cases, and a student’s perception of safety and protection can influence the likelihood of reporting a sexual assault. Indeed, many universities have indicated changes in rates of reports of assaults consistent with changes in campus policies regarding how such cases are handled. As such, although it is clear that alcohol often is involved in sexual assaults on college campuses, questions about the frequency and nature of such assaults remain.

## Spring Break and 21st Birthday Celebrations—Event-Specific Drinking Occasions

More college students drink, and drink more heavily, during specific celebratory events, such as spring break and 21st birthday celebrations, than during a typical week. Spring break is a roughly weeklong recess from school that takes place in the spring at colleges throughout the United States. While some students continue to work, travel home, or simply relax, others use the opportunity to travel to beaches and other party destinations. During spring break, approximately 42 percent of students get drunk on at least 1 day, 11 percent drink to the point of blacking out or passing out, 32 percent report hangovers, and 2 percent get into trouble with the police (Litt et al. 2013). Students with a history of binge drinking and those intending to get drunk tend to drink the heaviest (Patrick et al. 2013), suggesting that prevention efforts aimed at altering students’ intentions to get drunk while on spring break might lead to a reduction in peak drinking and the consequences that follow (Mallett et al. 2013). Interestingly, students who typically are light drinkers are more likely than those who typically are binge drinkers to experience consequences from excessive drinking during spring break (Lee et al. 2009).

In addition to spring break, 21st birthday celebrations are another event-specific opportunity for students to drink excessively. An estimated 4 out of 5 college students drink alcohol to celebrate their 21st birthdays (Rutledge et al., 2008) and many students drink more than they plan. Of 150 male and female college students surveyed about their intentions to drink during their upcoming 21st birthday celebrations, 68 percent consumed more than they anticipated while only 21 percent drank less and 11 percent were accurate. On average, males intended to consume 8.5 drinks but consumed 12.5, while females expected to drink 7 but had 9 (Brister et al., 2010). As with spring-break drinking, students with a history of binge drinking and those who intended to drink heavily on their 21st birthday consumed the most (Brister et al., 2011). In one study, roughly 12 percent of students reported consuming 21 or more drinks while celebrating, and one-third of females (35 percent) and nearly half of males (49 percent) reached estimated BACs above 0.25 percent (Rutledge et al., 2008). Such high levels of consumption substantially increase the odds of sexual assaults, fights, injuries, and death (Mallett et al., 2013). Research indicates that brief interventions conducted in the week leading up to the 21st birthday celebration can reduce levels of consumption and associated consequences, suggesting that the risks of experiencing alcohol related consequences stemming from 21st birthday celebrations could be partially mitigated through specifically timed prevention efforts (Neighbors et al. 2009, 2012).

## Summary

We have learned a considerable amount about the drinking habits of college students and the consequences that follow since NIAAA first reported on the matter in 1976. Surprisingly, drinking levels have remained relatively stable on and around college campuses over the last 30 years, with roughly two out of five male and female students engaging in excessive, or binge, drinking. Excessive drinking results in a wide range of consequences, including injuries, assaults, car crashes, memory blackouts, lower grades, sexual assaults, overdoses and death. Further, secondhand effects from excessive drinking place non–binge-drinking students at higher risk of injury, sexual assaults, and having their studying disrupted.

Estimates of the rates of alcohol use and related consequences are imperfect. Lack of knowledge of standard drink sizes and the effects of alcohol on memory formation all complicate the collection of accurate data from traditional self-report surveys. Underreporting of sexual assaults leads to difficulty in estimating the true extent of the problem. Lack of college identifiers in mortality records and the fact that alcohol levels are tested too infrequently in non–traffic-related deaths leaves uncertainty regarding the actual number of college students who die each year from alcohol-related causes. Similarly, college identifiers are not present in most crime reports and hospital reports.

Although it is beyond the scope of this review to examine efforts to prevent excessive drinking on college campuses, it should be noted that important strides have been made in this area (Carey et al. 2012). In addition, data from MTF suggest that levels of binge drinking are decreasing among 12th graders, particularly males. Hopefully, as our understanding of the nature of the problem continues to improve with better measurement strategies, improvements in prevention approaches combined with declines in precollege drinking will lead to reductions in both the levels of alcohol consumption by college students and the negative consequences that result.

## Footnotes

1 The ALDH2*2 allele results in decreased action by the enzyme acetaldehyde dehydrogenase, which is responsible for the breakdown of acetaldehyde. The accumulation of acetaldehyde after drinking alcohol leads to symptoms of acetaldehyde poisoning, such as facial flushing and increased heart and respiration rates.
